# Cardiac resynchronization therapy in patients with heart failure: systematic review

**DOI:** 10.1590/S1516-31802009000100009

**Published:** 2009-05-11

**Authors:** Hernani Pinto de Lemos, Álvaro Nagib Atallah

**Affiliations:** 1 MD, PhD. Researcher in the Brazilian Cochrane Center and Discipline of Evidence-Based Medicine of Universidade Federal de São Paulo - Escola Paulista de Medicina (Unifesp-EPM), São Paulo, Brazil.; 2 MD, PhD. Full professor and Head of the Discipline of Emergency Medicine and Evidence-Based Medicine of Universidade Federal de São Paulo - Escola Paulista de Medicina (Unifesp-EPM). Director of the Brazilian Cochrane Center and Scientific Director of Associação Paulista de Medicina (APM), São Paulo, Brazil.

**Keywords:** Heart failure, Heart conduction system, Bundle-branch block, Pacemaker, artificial, Mortality, Insuficiência cardíaca, Sistema de condução cardíaco, Bloqueio de ramo, Marca-passo artificial, Mortalidade

## Abstract

**CONTEXT AND OBJECTIVE:**

Cardiac resynchronization therapy (CRT) has emerged as the predominant electrical treatment strategy for patients on pharmacological therapy who present heart failure with wide QRS and low ejection fraction. The objective of this study was to investigate whether cardiac resynchronization therapy improved mortality and morbidity among patients with heart failure.

**METHODS:**

This was a systematic review using the Cochrane Collaboration’s methodology. The online search strategy included the Cochrane Library, Medline (Medical Literature Analysis and Retrieval System Online), Lilacs (Literatura Latino-Americana e do Caribe em Ciências da Saúde) and cardiology congresses from 1990 to 2006. The criteria for considering studies for this review were as follows:-types of studies: randomized controlled trials; types of interventions: cardiac resynchronization therapy compared with other therapies; types of participants: patients with heart failure with low ejection fraction and wide QRS; outcomes: death or hospitalization.

**RESULTS:**

Seven trials met the selection criteria. The risk of death due to congestive heart failure was nonsignificant: relative risk (RR), 0.79; 95% confidence interval (CI): 0.60 to 1.03. There was an absolute risk reduction of 4% in all-cause mortality for the experimental group [RR 0.70; CI: 0.60 to 0.83; number needed to treat (NNT) 25]; sudden cardiac death showed a statistically significant difference favoring the experimental group, with absolute risk reduction of 1% (CI: 0.46 to 0.96; RR 0.67; NNT 100). There was an absolute risk reduction of 9% for hospitalization due to heart failure (RR 0.64; CI: 0.50 to 0.80; NNT 11) in the experimental group.

**CONCLUSIONS:**

Patients receiving CRT had a significantly lower risk of hospitalization due to heart failure, but death rates due to heart failure were similar.

## RESUMO

**CONTEXTO E OBJETIVO:**

A terapia de ressincronização cardíaca (TRC) surgiu como a estratégia de tratamento elétrico predominante para pacientes com insuficiência cardíaca com QRS largo e baixa fração de ejeção. O objetivo foi investigar se a terapia de ressincronização cardíaca melhora a mortalidade e morbidade dos pacientes com insuficiência cardíaca.

**MÉTODOS:**

Esta é uma revisão sistemática que utilizou a metodologia da Colaboração Cochrane. A estratégia de busca eletrônica incluiu a Biblioteca Cochrane, Medline, Lilacs e congressos de cardiologia de 1990 a 2006. Os critérios de inclusão foram os seguintes: tipos de estudos: estudos clínicos randomizados; tipos de intervenções: terapia de ressincronização cardíaca comparada com outras terapias; tipos de participantes: pacientes com insuficiência cardíaca com baixa fração de ejeção e QRS largo; desfechos: mortalidade, hospitalização.

**RESULTADOS:**

Sete estudos foram incluídos. O risco de morte devida à insuficiência cardíaca congestiva foi insignificante: risco relativo (RR) = 0.79; intervalo de confiança (IC) de 95% = 0.60 a 1.03; houve redução de 4% do risco absoluto de mortalidade por todas as causas no grupo experimental [RR 0.70; IC: 0.60 a 0.83; número necessário para tratar (NNT) = 25]; morte cardíaca súbita mostrou ter diferença estatisticamente significante favorável ao grupo experimental, com redução de 1% do risco absoluto (IC: 0.46 a 0.96; RR 0.67; NNT= 100). Houve redução de 9% do risco absoluto de hospitalização devido a insuficiência cardíaca (RR 0.64; IC: 0.50 a 0.80; NNT =11) no grupo experimental.

**CONCLUSÕES:**

Pacientes em TRC tiveram risco de hospitalização por falência cardíaca significativamente mais baixo, mas os índices de mortalidade por insuficiência cardíaca foram semelhantes.

## INTRODUCTION

Although pharmacological therapies for congestive heart failure (CHF) have advanced over recent decades, morbidity and mortality have remained high. It is an often-quoted statistic that heart failure affects almost five million people in the United States alone, with about 500,000 new diagnosed cases per year.^1^^,^^2^ Heart failure is the leading cause of hospitalization among patients older than 65 years,^3^ and it is the only form of cardiovascular disease that is still increasing in prevalence. For advanced heart failure patients, angiotensin-converting enzyme (ACE) inhibitors were the first medication to achieve a reduction in mortality of 40% at six months.^4^ The second choice for pharmacological treatment of systolic dysfunction is beta-blockers.^5^ Angiotensin-receptor blockers have also been shown to reduce a combined outcome of morbidity and mortality.^6^^,^^7^ Spironolactone or eplerenone (aldosterone blockade) has demonstrated reductions in the relative risk of mortality, of 15% to 30% among patients with NYHA III/IV (New York Heart Association classification) and low ejection fraction.^8^ The non-pharmacological management consists mainly of lifestyle modification such as sodium restriction, and avoidance of non-steroidal anti-inflammatory drugs.^9^^,^^10^^,^^11^

In spite of everything, medical therapy and lifestyle modifications have been insufficient for treating heart failure patients. Mortality and hospitalization rates still need to be reduced. Disorders of the heart’s electrical transport can alter cardiac output, independent of the best pharmacological management. Delays in electrical conduction that occur in the presence of left bundle branch block, together with increased overall ventricular time due to delayed activation of the left free wall, results in mechanical dyssynchrony. Equally, this can happen because of traditional pacemaker implantation (right atrial and ventricular stimulation) with delayed activation of the left ventricle, thus generating ventricular dyssynchrony with alteration of the cardiac debit. This dyssynchrony does not have any systemic repercussion in normal hearts, but in an insufficient heart, the repercussion is significant and induces worsening of the heart failure. Cardiac resynchronization therapy by means of a multisite pacemaker offers pacing simultaneously in the right atrium, right ventricle and left ventricle. It is able to correct the dyssynchrony and improve both cardiac function and medical therapy.

The objective of this systematic review was to investigate whether cardiac resynchronization therapy improved mortality and reduced hospitalizations among patients with heart failure and low ejection fraction and wide QRS.

## METHODS

The type of study considered for this review was randomized controlled trials. The types of participants considered were patients with heart failure with low ejection fraction and QRS ≥ 120 msec, who were classified as NYHA II, III or IV. The type of intervention considered was the multisite pacemaker, in comparison with medical therapy or a univentricular pacemaker. The primary outcome considered was mortality and the secondary outcome was hospitalization due to heart failure.

The search strategy consisted of reviewing the databases of the Cochrane Library, Medical Literature Analysis and Retrieval System Online (Medline) and Literatura Latino-Americana e do Caribe em Ciências da Saúde (Lilacs), and cardiology congresses. The searches were restricted to the period from 1990 to 2006. There were no limitations on language or date of publication, or other possible restrictions.

The following keywords were accessed: “heart failure, congestive” or “heart failure, congestive/” or “heart failure, congestive/CO” or “heart failure, congestive/MO” or “heart failure, congestive/TH” [subject descriptor] and “cardiac pacemaker, artificial” or “cardiac pacemaker, artificial/” or “cardiac pacemaker, artificial/AE” or “cardiac pacemaker, artificial/UT” [subject descriptor] and “controlled clinical trial” or “meta-analysis” or “multicenter study” or “randomized controlled trial” [publication type].

The methodological quality of the selected trials was assessed using the criteria described in the Cochrane Handbook (Handbook 2004).^12^

**Figure 1.**
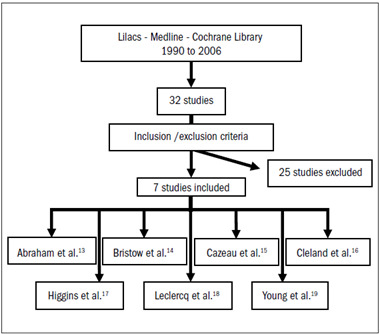
Methodological flow of search strategy.

The statistical analyses were performed using the RevMan computer software (Cochrane Center: available at: http://www.cochrane.org). Dichotomous outcomes were analyzed by calculating the relative risk (RR) for each trial, and the uncertainty in each result was expressed using confidence intervals (CI). When the overall results were significant, the number needed to treat (NNT) was calculated. The RR estimates were based on a random effects model (Figure 1).

## RESULTS

Seven randomized trials with 3164 patients met the inclusion criteria for this systematic review: Abraham et al.,^13^ Bristow et al.,^14^ Cazeau et al.,^15^ Cleland et al.,^16^ Higgins et al.,^17^ Leclercq et al.^18^ and Young et al.^19^ These were all published studies. The etiology of heart failure was unimportant for the patients recruited, but acute ischemia, uncorrectable valve disorders and hypertrophic or restrictive cardiomyopathy were excluded from all these trials. One other trial in which the control group consisted of patients with a univentricular pacemaker was restricted to patients with atrial fibrillation.^20^

QRS width was a criterion for all the included trials: three trials specified ≥ 120 msec;^14^^,^^16^^,^^17^ two trials ≥ 130 msec;^13^^,^^19^ one trial ≥ 150 msec;^15^ and one trial ≥ 200 msec.^18^ No trials recruited patients with an ejection fraction > 40%. One trial included NYHA class II.^17^ Bristow’s study^14^ had three arms, comparing optimal pharmacological therapy, cardiac resynchronization therapy and cardiac resynchronization therapy with cardioverter defibrillator. The follow-up for each study ranged from two months to 18 months.

All of these studies had good methodological quality. However, their inclusion criteria and choice of control group were heterogeneous.

### Mortality

**Figure 2.**
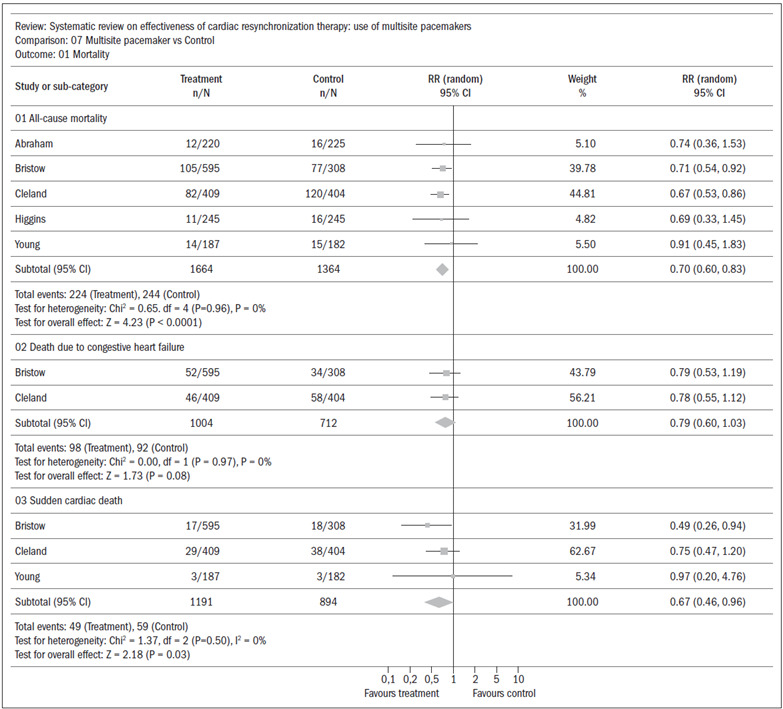
Systematic review: meta-analysis on mortality.

This outcome was considered in three ways:

1. All-causes mortality: five studies^13^^,^^14^^,^^16^^,^^17^^,^^19^ presented data that were put into a meta-analysis that found a statistical difference favoring the experimental group, with an absolute risk reduction of 4%; CI: 0.60 to 0.83; RR 0.65; and NNT 25 (Figure 2).
2. Death due to congestive heart failure: two studies^14^^,^^16^ presented data that were put into a meta-analysis that did not find any statistical difference between the groups (CI: 0.60 to 1.03; RR 0.79) (Figure 2).
3. Sudden cardiac death: three studies^14^^,^^16^^,^^19^ were included in a meta-analysis that found a slight but statistically significant difference favoring the experimental group, with an absolute risk reduction of 1% (CI: 046 to 0.96; RR 0.67) (Figure 2).

### Hospitalization due to heart failure

**Figure 3.**
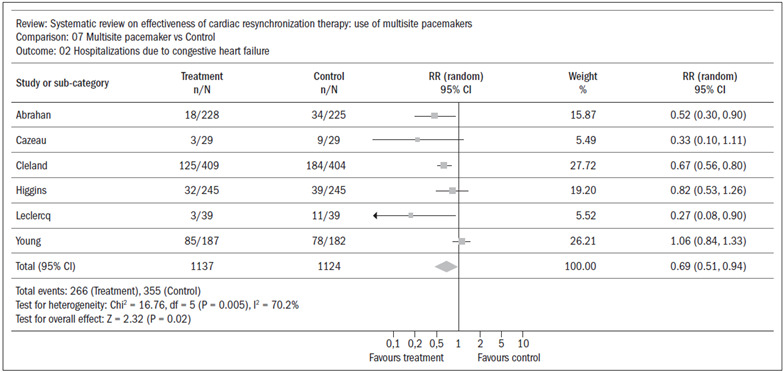
Systematic review: meta-analysis on hospitalization; I^2^= 70.2%.

**Figure 4.**
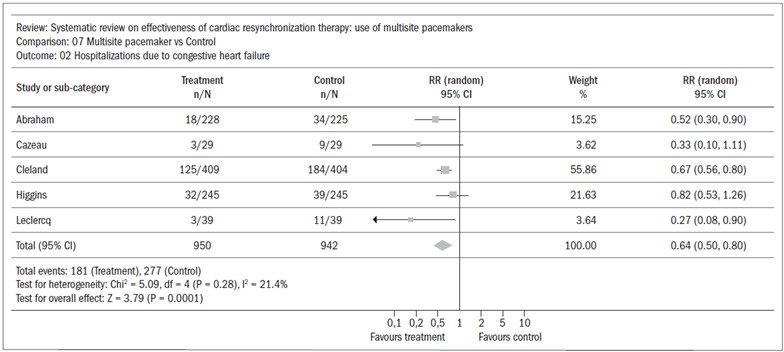
Systematic review: meta-analysis on hospitalization without Young’s study;^19^ I^2^ = 21.4%.

Six studies^13^^,^^15^^,^^16^^,^^17^^,^^18^^,^^19^ were put into a meta-analysis that found a reduction in the absolute risk of hospitalization of 9%, favoring the experimental group (CI: 0.51 to 0.94; RR 0.69; NNT 11) (Figure 3). In this meta-analysis there was statistical heterogeneity, which was demonstrated by I^2^ > 50% (I^2^ = 70.2%). Visually, in Figure 3, the study by Young et al.^19^ is separate from the others. We removed this study and redid the meta-analysis, and the statistical heterogeneity disappeared, such that I^2^ = 21.4% (Figure 4). This alteration did not modify the result from the meta-analysis, which continued to favor the experimental group. We looked for a source of clinical heterogeneity to explain this finding and we saw that, on the whole, the disease was more severe among the patients in Young’s study^19^ (i.e. they had an indication for an implantable cardiac defibrillator), with less chance of gaining the morphometric remodeling benefits that might be associated with cardiac resynchronization. Other secondary outcomes (quality of life, six-minute walking distance test and functional class) were analyzed in this systematic review, but these merely reflected the morbidity that was more broadly expressed through the need for hospitalization.

## DISCUSSION

When cardiac resynchronization therapy was added to other forms of medical therapy, the morbidity rate decreased, i.e. there was an effective improvement in patients’ conditions, with a 9% reduction in hospitalization due to heart failure. The primary and main outcome of this review, i.e. mortality, was expressed in several ways: all causes, due to heart failure and due to sudden cardiac death. Among these, mortality due to heart failure was the main expression of this study. However, unfortunately, this measurement could only be extracted from two studies, for which the meta-analysis did not show statistical significance. All-cause mortality was extracted from five studies and showed an absolute risk reduction of 6% favoring the experimental group, while the other form of expression for mortality (sudden cardiac death) was extracted from three studies, and showed statistical significance for the experimental group with an absolute risk reduction of 1%.

Since the intervention proposed in this study addresses heart failure, it has to be inferred that heart resynchronization by means of a multisite pacemaker is ineffective under the circumstances presented in this review, with regard to modifying the progression to death. However, it is difficult to interpret this in daily medical practice, since sudden death could occur in a case of progressive heart failure without hospitalization that is oligosymptomatic because of excessive physical self-restriction, while a case of supposed progressive heart failure with hospitalization could be the result of significant acute electrical disturbances caused by drug use or non-use, among patients who were previously stable. This shows that biventricular stimulation improves the myocardial contractile synchrony, which consequently gives rise to hemodynamic improvement but without any change to mortality. On the other hand, univentricular stimulation causes myocardial contractile dyssynchrony that worsens the heart failure and increases the numbers of hospitalizations.

Our data in this systematic review on mortality are concordant with the data in a previous systematic review,^20^ which found statistically significant data favoring the experimental group with regard to all-causes mortality (reduction by 21%; RR 0.79; CI: 0.66 to 0.96), but without statistical significance for death due to congestive heart failure (RR 0.60; CI: 0.36 to 1.01). For the endpoint of hospitalization due to heart failure, the meta-analysis of the previous review did not demonstrate any statistically significant difference for any group (CI: 0.41 to 1.12), although in the results the author emphasized that they were reduced in the experimental group by 32%. One unpublished study was included in that outcome (RD-CHF^21^). Another study^19^ was put into the meta-analysis of the previous review^20^ but with an inexplicably different numbers of participants: 554 in total, of which 272 in the experimental group and 282 in the control group (the published version of the Miracle ICD study has, however 369 participants: 187 in the experimental group and 182 in the control group^19^).

Another review conducted on four studies^22^ (three unpublished studies with data from the internet and one published study^15^) resulted in a meta-analysis favoring multisite pacemakers for reducing death due to congestive heart failure (odds ratio, OR: 0.49; CI: 0.25 to 0.93), but without statistical significance for all-causes mortality (OR 0.77; CI: 0.51 to 1.18).

In a systematic review published in 2006,^23^ the meta-analysis result regarding the endpoints of mortality due to congestive heart failure and sudden death differed from ours, in that it was statistically favorable towards the experimental group regarding mortality due to congestive heart failure (OR 0.62; CI: 0.45 to 0.84), although without any statistically significant difference regarding sudden death (OR 1.04; CI: 0.73 to 1.22). For the outcome of mortality due to congestive heart failure, the meta-analysis was conducted on five studies, of which two had zero mortality. Among the other three studies, one^13^ was not included in our meta-analysis because it did not present objective data for extraction regarding this endpoint. All-cause mortality was significantly reduced, by 29% (OR 0.71; CI: 0.57 to 0.88).

Our study has potential differences with other systematic reviews because we only used data from studies reported in the format of journal articles. We did not use data coming from internal studies providing information for the pharmaceutical industry or from sources other than articles published in medical journals. Our systematic review did not include any data from unpublished studies. All the data from the studies included in this systematic review were extracted by the present authors from published articles, as shown by the respective references.

## CONCLUSION

The use of multisite pacemakers with cardiac resynchronization therapy was associated with functional improvements for patients with heart failure, thereby decreasing the rate of hospitalization due to heart failure. From the results of this systematic review, there is no evidence that cardiac resynchronization therapy reduces specific death due to congestive heart failure.
